# LEM-UNet: an edge-guided network for 3D multimodal images segmentation in focal cortical dysplasia

**DOI:** 10.3389/fnins.2025.1634606

**Published:** 2025-10-08

**Authors:** Qiunan Li, Hao Yu, Manli Zhang, Xiaotong Yuan, Lixin Cai, Guixia Kang

**Affiliations:** ^1^School of Information and Communication Engineering, Beijing University of Posts and Telecommunications, Beijing, China; ^2^Pediatric Epilepsy Center, Peking University First Hospital, Beijing, China

**Keywords:** focal cortical dysplasia, multimodal medical imaging, deep learning, edge information, medical image segmentation

## Abstract

**Introduction:**

Focal cortical dysplasia (FCD) is one of the common causes of refractory epilepsy. The subtle and indistinct edge of FCD lesions pose considerable challenges for accurate lesion localization. Therefore, we propose an edge guided segmentation network based on Laplacian pyramid to improve the localization performance of FCD lesions.

**Methods:**

This is a retrospective study evaluated on two independent datasets. The proposed Laplacian Edge Mix UNet (LEM-UNet) builds upon the MedNeXt baseline and incorporates the Laplacian Edge Attention (LEA) block and the Multi-strategy Feature Fusion (MFF) block. LEA block captures lesion details and edge information during the encoding phase by integrating Laplacian pyramid feature maps with an attention mechanism, while MFF block fuses edge features with high level features during the decoding phase.

**Results:**

The model's performance was assessed through 5-fold cross-validation across both Open and Private Datasets, demonstrating superior performance. The average Dice Coefficient achieved 0.452 and 0.597 on the Open and Private Datasets, respectively, representing improvements of 2.40% and 2.90% compared to the baseline model.

**Discussion:**

The results demonstrate the importance of focusing on lesion edge in the FCD segmentation task. The integration of the Laplacian pyramid enhances the mode's ability to capture lesions with blurred edge and subtle features. LEM-UNet exhibits significant advantages over current FCD segmentation algorithms. The source code and pre trained model weights are available at https://github.com/simplify403/LEM-UNet.

## 1 Introduction

Epilepsy is a prevalent chronic neurological disorder characterized by the abrupt, abnormal discharge of brain neurons (Fisher et al., 2005). Most epilepsy patients can control seizures using medications, but one-third of patients develop drug-resistant epilepsy (DRE). Prolonged epileptic seizures can lead to severe consequences such as brain damage, decreased quality of life, and even premature death (Avakyan et al., 2017). Focal cortical dysplasia (FCD), a malformation of cortical development, is the most common cause of DRE (Kabat and Król, 2012). Clinically, an effective treatment for patients with DRE is surgical resection of FCD lesions. About 70% of patients experience seizure relief after surgery (Wagstyl et al., 2022). A favorable outcome is associated with the complete resection of FCD lesions (Willard et al., 2022).

**Figure 1:**
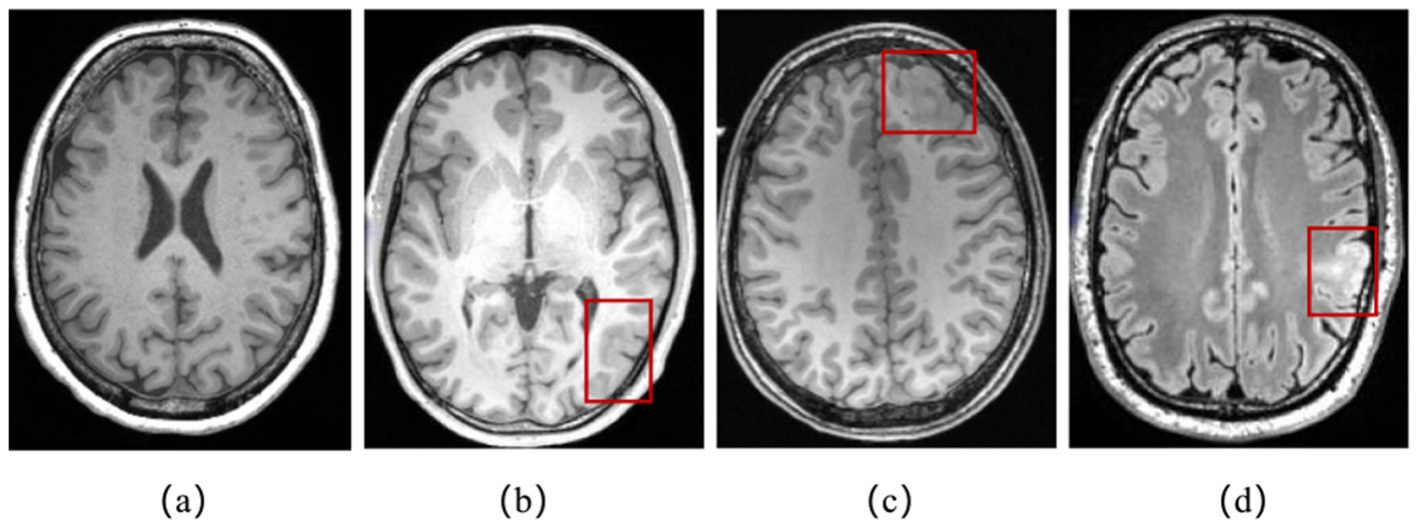
**(a)** A T1-weighted MRI slice of normal brain; **(b)** abnormal gray matter thickening in a T1-weighted MRI slice in red box; **(c)** blurred junction of gray and white matter in a T1-weighted MRI slice in red box; **(d)** increased signal intensity in FLAIR in red box.

In clinical diagnosis, physicians can identify FCD lesions by analyzing neuroimaging results. Magnetic Resonance Imaging (MRI) is a medical imaging technique that uses the principles of nuclear magnetic resonance to produce detailed images of the internal structures of the human body. On MRI scans, characteristic manifestations of FCD include increased cortical thickness, blurring of the gray/white matter junction, the presence of a transmantle sign, abnormal cortical folding pattern, and increased signal intensity on FLAIR/T2-weighted MRI (Urbach et al., 2022) as depicted in Figure 1. Positron Emission Tomography (PET) is a nuclear medicine imaging technique used to observe biological processes and functional activities within the human body. Between seizures, PET images using fluorodeoxyglucose show hypometabolism in areas of gray matter tissue that are associated with the epileptogenic region (Baete et al., 2004). However, the identification of FCD lesions in clinical practice remains a formidable challenge. Firstly, FCD lesions are often subtle, exhibit diverse morphological features, and possess poorly defined edge, making them difficult to detect through routine visual inspection. Secondly, the analysis of high-resolution 3D imaging datasets is time-consuming, requires specialized expertise, and can be subjective in certain cases. Therefore, there is an urgent need for precise and efficient computational techniques to assist in the localization of FCD lesions.

In the early stages, the task of FCD lesion detection primarily focused on the extraction of cortical morphological features, with the main approaches including voxel-based morphometry (Mechelli et al., 2005) and surface-based morphometry (Thesen et al., 2011). The extracted voluminous cortical morphological features were then input into machine learning models to distinguish between normal and pathological tissues, such as (David et al. 2021); (Jin et al. 2018); and (Hong et al. 2014). However, these methods exhibit several limitations: the computation of features is complex and extremely time-consuming, and some basic features are insufficient to accurately differentiate FCD lesions.

Currently, deep learning-based models have achieved remarkable success in various medical imaging tasks, significantly advancing disease detection and diagnosis. Convolutional Neural Networks (CNNs) are the most widely used model for the detection of FCD lesions, with a lot of work is based on it, such as (Gill et al. 2021) and (Wang et al. 2020). Enhanced model designs have led to improved detection performance. (Dev et al. 2019) trained a U-Net using 2D slices derived from 3D FLAIR-weighted sequence images. (Niyas et al. 2021) proposed a 3D Res-UNet model for segmenting FCD lesions from MRI volumes, leveraging inter-slice information from 3D MRI data to achieve superior segmentation performance. (Thomas et al. 2020) introduced a Multi-Res-Attention UNet to address the significant semantic gap in feature mapping caused by long-range connections between the encoder and decoder layers, resulting in a higher FCD detection rate. The nnU-Net framework has been proposed as a self-adaptive solution for diverse medical image segmentation tasks (Isensee et al., 2018). (Spitzer et al. 2023) developed a graph-based nnU-Net for segmenting FCD lesions using surface-based cortical data, achieving a 22%–27% improvement in specificity compared to baseline methods. Zhang S. et al. (2024) employed a 3D full-resolution nnU-Net for automatic lesion segmentation, demonstrating strong performance in FCD lesion detection with a sensitivity of 0.73. Zhang X. et al. (2024) integrated multi-scale transformers into CNN-based encoding and decoding structures to overcome the limitations of local receptive fields in CNN models and successfully identified lesions in 82.4% of patients.

The aforementioned methods have significantly contributed to the detection of FCD lesions. However, traditional methods based on manual extraction of cortical morphological features combined with machine learning exhibit several limitations: (1) feature extraction is computationally complex and time-consuming, (2) basic features are insufficient to fully capture lesion characteristics. Transformer-based models, while powerful, demand substantial computational resources and are challenging to train on small datasets. CNN-based models have demonstrated significant potential in the field of lesion segmentation due to their excellent local perception capabilities. However, they still exhibit limitations when applied to FCD lesion segmentation tasks. FCD lesions are characterized by blurred edge, making it challenging for networks to accurately capture and identify edges and subtle lesions. Integrating edge detection methods into convolutional models opens new avenues to address these challenges. In other medical image segmentation tasks, models incorporating edge detection methods have shown exceptional performance, as seen in (Zhu et al. 2023) and (Lu et al. 2023). However, there is a paucity of relevant research in the task of FCD lesion segmentation.

To address these clinical needs and the shortcomings of existing algorithms, this study proposes an edge information-guided FCD lesion segmentation model. Based on the MedNext (Roy et al., 2023) network architecture, the main contributions of this paper are as follows:

1. We propose a Laplacian edge attention block to extract edge features from the T1 modality and integrate them with attention mechanisms, enabling spatial localization from global edges to FCD lesion edges.
2. We propose a multi-strategy feature fusion block to effectively combine high-frequency edge information with network decoding information.

## 2 Materials and methods

### 2.1 Materials

Our network was evaluated on two distinct datasets: the Open Dataset, provided by the Department of Epileptology at the University Hospital Bonn (Schuch et al., 2023) and our proprietary dataset, referred to as the Private Dataset. More detailed information on the two datasets is shown in Table 1 and the brief introduction of the two datasets is as follows.

**Table 1:** Subjects characteristics of FCD.

**Characteristics**	**Private dataset**	**Open dataset**
**Number**	**125**	**78**
Age of surgery(years, mean ± std)	5.98 ± 4.15	6.30 ± 2.48
Sex (male:female)	78:47	44:34
Hemisphere (left:right)	60:65	41:37
Undergoing surgery	125	46
**Pathology**		
FCD IA	3	0
FCD IB	10	0
FCD IIA	31	15
FCD IIB	52	31
FCD IIIB	1	0
MCD	23	0
TSC	2	0
Gliosis	1	0
MOGHE	2	0
NA	0	32
**One year outcome**		
Seizure-free	118	34
Not seizure-free	7	8
NA	0	36

std, standard deviation; FCD, focal cortical dysplasia; MCD, malformation of cortical development; TSC, tuberous sclerosis complex; MOGHE, mild malformation of cortical development with oligodendroglial hyperplasia; NA, not applicable; Pathology classifications are based on the International League Against Epilepsy (ILAE) criteria recommendations for the neuropathologic workup of epilepsy surgery brain tissue.

**Open dataset:** Open dataset consists of 85 people with FCD type II and 85 control persons. Data for each patient include preoperative T1, preoperative FLAIR and lesion ground truth. Lesion ground truth was annotated on FLAIR images by two neurologists. Because 7 patients underwent a FLAIR sequence with thick slices, we excluded these data and ended up with 78 3D cases.

**Private dataset:** Private dataset consists of 125 patients. Among them, 41.6% of the patients were diagnosed with FCD IIB pathologically, 24.8% with FCD IIA, and 18.4% with other types of malformation of cortical development, while the remaining small proportion was attributed to other etiological causes. Each patient contains preoperative 3-T T1, FLAIR, FDG-PET images, and lesion ground truth. Lesion ground truth was annotated on T1 images by the physician. This study has obtained approval from the Ethics Committee of Peking University First Hospital. All participants provided written informed consent for us to use their data for research purposes.

### 2.2 Methods

#### 2.2.1 Model architecture

Before detailing our method, we provide a concise overview of the MedNeXt network. MedNeXt enhances the nnU-Net framework by introducing several specialized modules: MedNeXt Block, MedNeXt 2 × down Block, MedNeXt 2 × up Block, Stem Block and Output/Deep Supervision Block. Our approach adopts the MedNeXt network, adhering to the architectural components outlined in the original paper.

**Figure 2:**
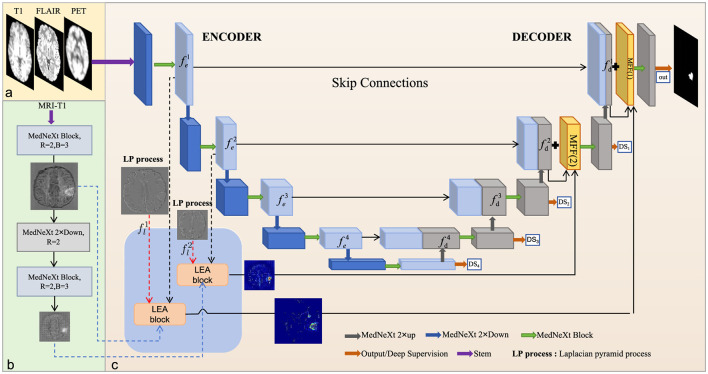
The overall framework of the proposed methodology. **(a)** Inputs to the model; **(b)** encoding of the T1 image; **(c)** the architecture of LEM-UNet. The LEA and MFF blocks are integrated into the first two encoding layers and the last two decoding layers, respectively. The three-colored dashed data stream denotes the three inputs to the LEA block. The dark blue blocks represent downsampling or stem results, the gray blocks represent the results after upsampling or MedNeXt block, and the light blue block represents the results after MedNeXt block.

The proposed network architecture, illustrated in Figure 2, comprises an encoder-decoder model, Laplacian edge attention blocks, and multi-strategy feature fusion blocks. The encoder-decoder model, derived from MedNeXt, supports composite scaling in depth, width, and receptive field to effectively extract contextual and high-level features from multimodal images. The Laplacian edge attention block integrates Laplacian pyramid features with encoded network features to enhance edge representation, directing the network's focus toward lesion edge. The multi-strategy feature fusion blocks combine edge features with decoded network features, employing diverse fusion strategies across decoding layers to retain edge information from shallow features. The subsequent sections elaborate on the structure and efficacy of the Laplacian edge attention block and the multi-strategy feature fusion block.

##### 2.2.1.1 Laplacian edge attention block

The segmentation of FCD lesions is particularly challenging due to their ambiguous edge. The LEA block addresses this by enhancing edge information, enabling the network to better focus on lesion edge and thereby improving segmentation performance. Since edge features are inherently shallow, the LEA block is applied to the first and second layers of the encoding process to effectively capture texture details of lesion edges.

**Figure 3:**
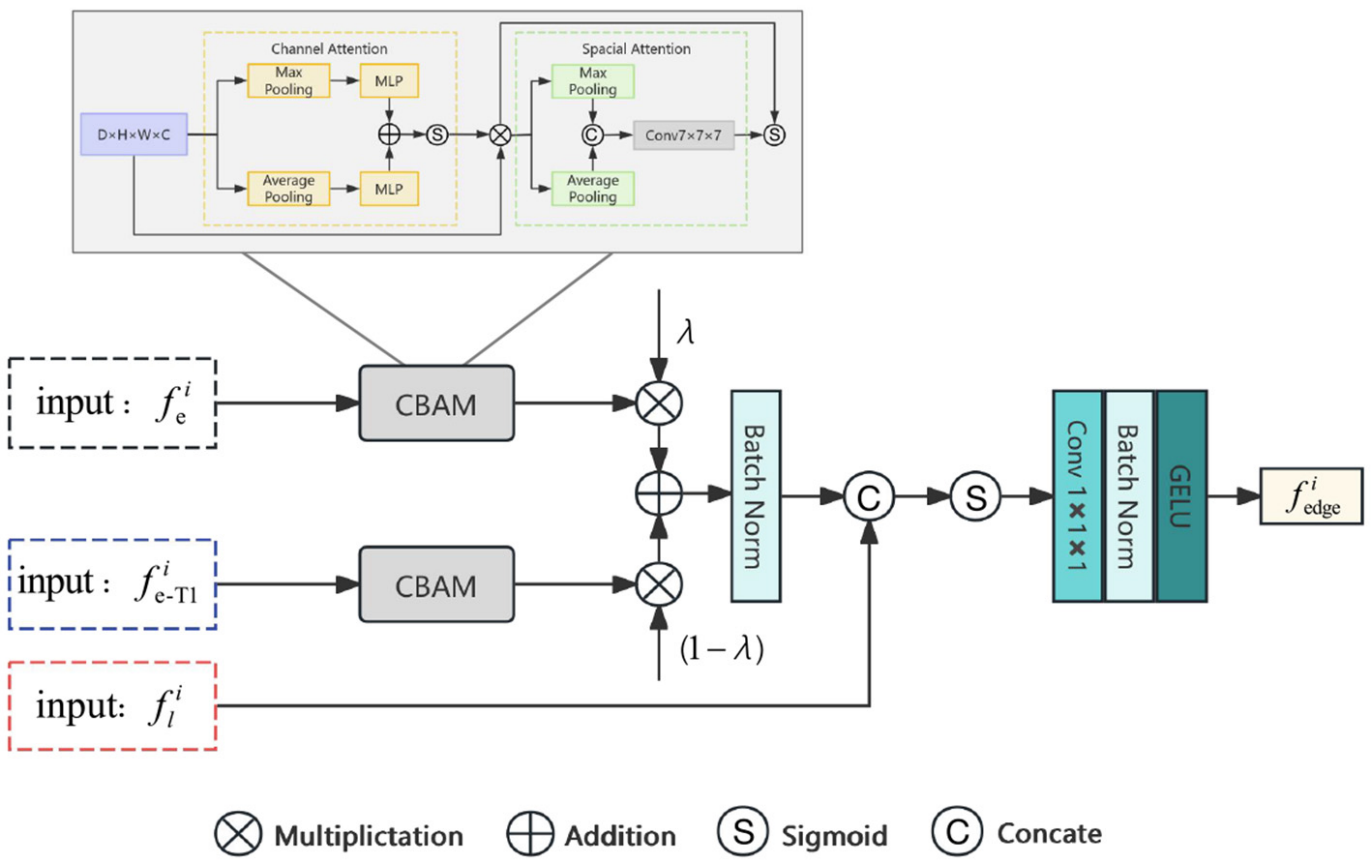
The architecture of Laplacian Edge Attention (LEA) block.

The LEA block proposed in this paper is shown in Figure 3. Each LEA block receives three inputs: encoded feature $f_{e}^{i}$ from the main network branch, encoded feature $f_{e-T1}^{i}$ from the T1 branch, and high-frequency features $f_{l}^{i}$ from the Laplacian pyramid. These features generate an output feature map $f_{edge}^{i}$ after being processed by the LEA block, which then enters the subsequent network for feature fusion processing. The following section will detail the inputs of the LEA block and its specific processing methods.

###### 2.2.1.1.1 Input of LEA block

The input of LEA block consists of three features.

- Encoded feature $f_{e}^{i}$: Our backbone network continues to use the MedNext encoding-decoding model to process multimodal data, representing the feature maps extracted after MedNext block as $f_{e}^{i}\in {\mathbb{R}}^{C_{i}\times D_{i}\times H_{i}\times W_{i}}$. To obtain rich shallow-level features, the first and second layer feature maps after MedNext processing are used, namely $f_{e}^{1}\in {\mathbb{R}}^{C\times D\times H\times W}$ and $f_{e}^{2}\in {\mathbb{R}}^{2C\times \frac{D}{2}\times \frac{H}{2}\times \frac{W}{2}}$, as shown by the black dotted data stream in Figure 2.
- Encoded feature $f_{e-T1}^{i}$ from branch T1: The T1-weighted MRI contains rich tissue texture and structural information. We individually extracted the characteristics, still using the MedNext network blocks, and obtained characteristics $f_{e-T1}^{1}\in {\mathbb{R}}^{C\times D\times H\times W}$ and $f_{e-T1}^{2}\in {\mathbb{R}}^{2C\times \frac{D}{2}\times \frac{H}{2}\times \frac{W}{2}}$, as shown by the blue dotted data stream in Figure 2. Parameters *R* and *B* refer to paper (Roy et al., 2023).
- High frequency features $f_{l}^{i}$: The Laplacian pyramid is a multiresolution image representation method based on the Gaussian pyramid, capable of capturing edges and detail information of images at different scales (Burt and Adelson, 1987). In this paper, this classic edge detection technique is selected to highlight the high-frequency edge features and the detail features of the images. In the LEA block, T1-weighted MRI is used as input to construct the Laplacian pyramid, as T1-weighted MRI provides rich texture information and clear anatomical structures. The specific calculation can be seen in Equation 1.

(1) $$\begin{array}{l} N_{k}=N,ifk=0,\\ N_{k}=d\left(gs\left(N_{k-1}\right)\right),ifk\geq 1,\\ L_{k}=N_{k}-up\left(N_{k+1}\right), \end{array}$$

where *N* represents the input image, *gs* represents the convolution operator with Gaussian filter, *d* is the 2 × downsampling operation, and *up* is the 2 × linear interpolation upsampling. We denote $f_{l}^{i}=L_{i}\left(N\right)$. As the image scale decreases, due to multiple processes of Gaussian filtering, upsampling, and downsampling, the high-frequency details in the feature maps significantly decrease. Therefore, the first and second layers of the Laplacian pyramid feature $f_{l}^{1}\in {\mathbb{R}}^{1\times D\times H\times W}$and $f_{l}^{2}\in {\mathbb{R}}^{1\times \frac{D}{2}\times \frac{H}{2}\times \frac{W}{2}}$ are selected as inputs for the LEA block, as shown by the red dotted data stream in Figure 2.

###### 2.2.1.1.2 Procedure of LEA block

At the *i*^*th*^ layer, the LEA block receives the three inputs mentioned above. At first, $f_{e}^{i}$ and $f_{e-T1}^{i}$ are each passed through a Convolutional Block Attention Module (CBAM) (Woo et al., 2018). The CBAM primarily consists of two components: channel attention and spatial attention, which serve to emphasize useful channel features and important spatial locations, respectively. Subsequently, the weights of the feature maps are adjusted to enhance the representation of salient features and $f_{map}^{i}$ is obtained. The process is shown in Equation 2.


(2) $$\begin{array}{l} \beta =1-\text{λ},\\ f_{map}^{i}=BN\left(CBAM\left(f_{e}^{i}\right)\times \text{λ}+CBAM\left(f_{e-T1}^{i}\right)\times \beta \right), \end{array}$$

where *BN* is the batch normalization, λ is a learnable parameter, with the purpose of adjusting the weights of $f_{e}^{i}$ and $f_{e-T1}^{i}$, allowing the network to autonomously learn the complementary information between this two features.

The high frequency features $f_{l}^{i}$ correspond to the overall edge information and texture features of the original image. In order to obtain edge information and detailed features related to the FCD lesions, we fuse $f_{l}^{i}$ and $f_{map}^{i}$, further screening the features to ensure that the edges of the FCD lesions are highlighted, and finally obtain the edge attention feature $f_{edge}^{i}$, characterized as follows Equation 3.


(3) $$\begin{array}{l} map^{i}=\sigma \left(con\left[f_{l}^{i},f_{map}^{i}\right]\right),\\ f_{edge}^{i}=g\left(BN\left(Cov\left(map^{i}\right)\right)\right). \end{array}$$

In this context, the symbol σ signifies the sigmoid function, *Cov* signifies the 1 × 1 × 1 convolution, *con* is Concatenation operation and the symbol *g* represents GELU function.

Through the LEA block, we obtain the edge attention features for the first and second layers, denoted $f_{edge}^{1}\in {\mathbb{R}}^{C\times D\times H\times W}$ and $f_{edge}^{2}\in {\mathbb{R}}^{2C\times \frac{D}{2}\times \frac{H}{2}\times \frac{W}{2}}$, respectively. These features are then inputted into the feature fusion block, where they are combined with the backbone network to achieve better segmentation performance.

##### 2.2.1.2 Multi-strategy feature fusion block

**Figure 4:**
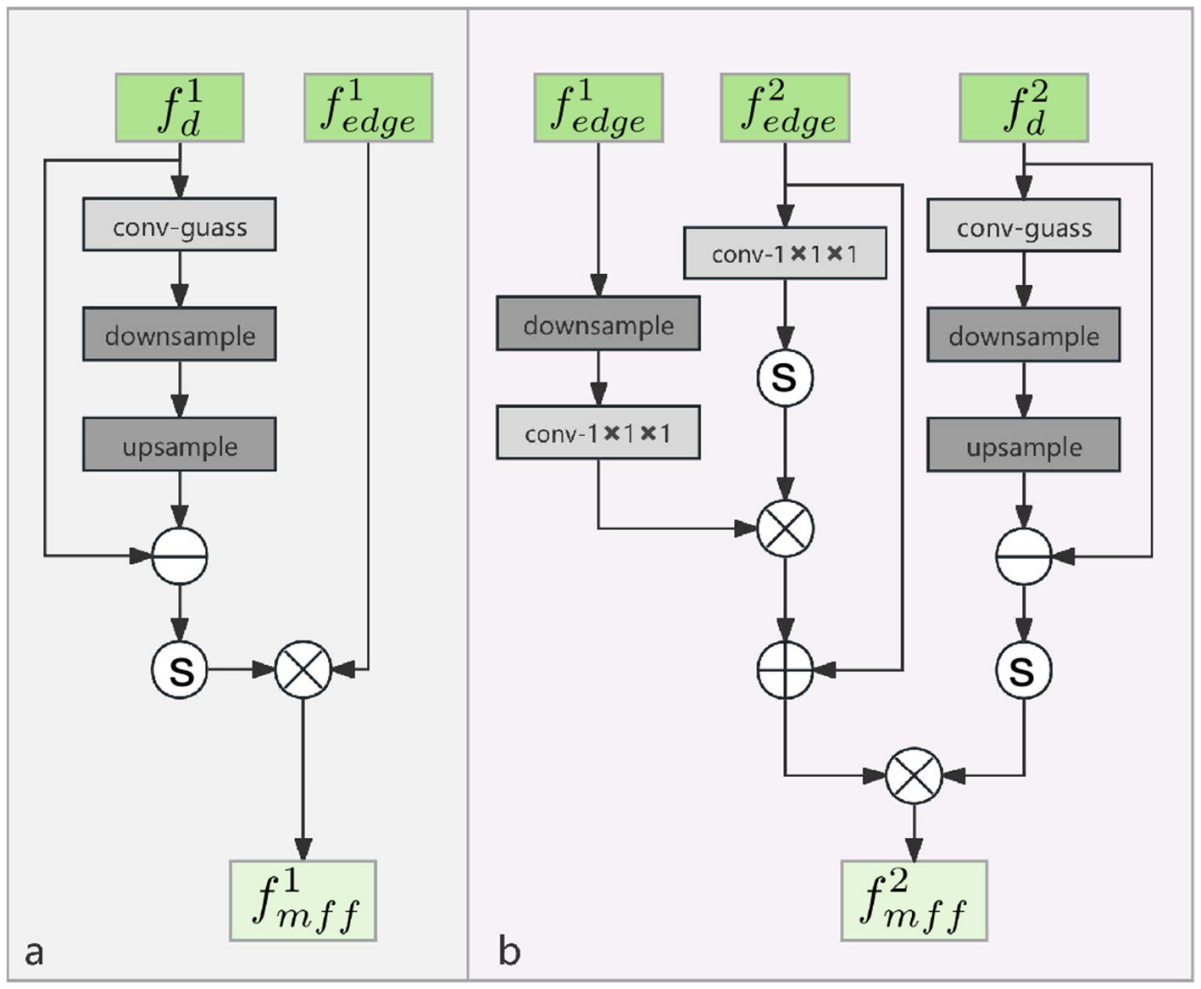
The architecture of multi-strategy feature fusion (MFF) block. **(a)** MFF(1); **(b)** MFF(2).

Multi-strategy feature fusion (MFF) block is shown in Figure 4. Our backbone network undergoes four downsampling and four upsampling operations. The upsampling features are now denoted as $f_{d}^{i}$, with the last upsampling result denoted as $f_{d}^{1}$ and the penultimate upsampling result as $f_{d}^{2}$. Each MFF block accepts two inputs: $f_{d}^{i}$ and $f_{edge}^{i}$, employing different fusion strategies at different decoder layers to maximize the use of edge information of the injury obtained from the LEA block. Define the output features of MFF block as $f_{mff}^{i}$.

In MFF (1), the inputs are processed as shown in Equation 4.


(4) $$\begin{array}{l} M^{i}=f_{d}^{i}-up\left(d\left(gs\left(f_{d}^{i}\right)\right)\right),\\ f_{mff}^{1}=\sigma \left(M^{1}\right)\times f_{edge}^{1}, \end{array}$$

where *M*^*i*^ represents the high-frequency information obtained from the decoded feature $f_{d}^{i}$ and it is designed to further suppress noise in the output of the LEA block. In MFF (2), the inputs are processed as shown in Equation 5.


(5) $$\begin{array}{l} I=\sigma (Cov(f_{edge}^{2}),\\ f_{mff}^{2}=\sigma (M^{2})\times (f_{edge}^{2}+Cov(d(f_{edge}^{1}))\times I). \end{array}$$

We employ distinct fusion strategies in MFF block. The encoded features contain rich texture information in the shallow layers, and as the network deepens, it increasingly focuses on complex high-level features and abstract representations. Similarly, in the Laplacian pyramid, with the increasing number of sampling iterations, the detail information in the feature maps is progressively lost.

Therefore, during the feature fusion phase, the edge information rich feature $f_{edge}^{1}$ undergoes a 2 × downsampling and is fed into the subsequent layer for further processing. The purpose of this operation is to make full use of the edge information from the shallow layers of the network and to minimize the loss of information. Finally, the fused feature $f_{mff}^{i}$ from the encoding layer *i*^*th*^ is added to $f_{d}^{i}$ for decoding.

#### 2.2.2 Loss function

We employ a combination of Dice loss L_*Dice*_ and Cross-Entropy loss L_*CE*_ at the training stage of our network. It can be defined as follows in Equation 6.


(6) $$\begin{array}{l} \begin{array}{c} L=\overset{D}{\underset{i=1}{\sum }}w_{dice}^{i}*L_{Dice}\left(f_{DS}^{i},f^{i}\right)+w_{ce}^{i}*L_{CE}\left(f_{DS}^{i},f^{i}\right), \end{array} \end{array}$$

where *D* denotes the total number of layers, as we use deep supervision in our network. $w_{dice}^{i}$ and $w_{ce}^{i}$ are the weights of L_*Dice*_ and L_*CE*_ at the *i*^*th*^ decoding layer. $f_{DS}^{i}$ is predicted feature map at the *i*^*th*^ decoding layer. *f*^*i*^ is Ground Truth (GT) at the *i*^*th*^ layer.

#### 2.2.3 Evaluation metrics

To quantitatively compare our method with others, we employ five evaluation metrics.

Dice Coefficient (DC) is used to measure the overlap between the predicted segmentation and the ground truth. To assess individual performance, we set the DC thresholds at 0.0 and 0.22. A DC score greater than 0.0, the so-called “one voxel overlap,” has been used in other FCD detection studies (David et al., 2021; Spitzer et al., 2022; Gill et al., 2021), and a higher threshold of 0.22 has been shown to be effective in reproducing the precise positioning performance of expert raters (Walger et al., 2024). Precision (Pre) represents the proportion of samples that are actually positive among those predicted as positive by the model and reflects the accuracy of the positive predictions of the model. Recall (Rec) indicates the proportion of actual positive samples that are correctly predicted as positive by the model and reflects the model's coverage ability in the positive class. Intersection over Union (IoU) is used to measure the degree of overlap between the result of the segmentation and the ground truth. They are defined as Equation 7.


(7) $$\begin{array}{l} DC(G,P)=2*\frac{\left|G\cap P\right|}{\left|G|\cup |P\right|}=2*\frac{G\cdot P}{G^{2}+P^{2}},\\ \;Pre=\frac{TP}{TP+FP},\\ \;Rec=\frac{TP}{TP+FN}, \end{array}$$

$$\begin{array}{c} IoU=\frac{G\cap P}{G\cup P},\\ Detection=\frac{N_{d}}{N_{a}}*100\%, \end{array}$$

where *G* and *P* denote the GT and the predicted results of the model, respectively. *TP* refers to the number of true positive voxels, *FP* refers to the number of false positive voxels and *FN* denotes the false negative voxels. *N*_*d*_ refers to the number of individuals with a dice value higher than the threshold of 0.0 or 0.22, while *N*_*a*_ denotes the total number of individuals. The higher the above metrics, the better the model performance.

Hausdorff Distance (HD) is a measure of similarity or difference between two sets of points, especially the edge difference between two shapes. *HD*_95_ uses a 95% quantile and is more robust, while HD is susceptible to outliers. A lower value *HD*_95_ indicates a higher degree of similarity and less difference between the two sets of points. *HD*_95_ is defined as Equation 8.


(8) $$\begin{array}{l} HD_{95}\left(G,P\right)=max\left\{d_{95}\left(G,P\right),d_{95}\left(P,G\right)\right\} \end{array}$$

Our experiment uses a five-fold cross-validation, so the above metrics are averaged.

#### 2.2.4 Comparison models

We included a variety of comparison models, namely: UNETR++ (Shaker et al., 2024), CoTr (Xie et al., 2021), nnFormer (Zhou et al., 2023), Res-Unet (Diakogiannis et al., 2020), and nnUNet. UNETR++, nnFormer, and CoTr represent successful integrations of CNNs and transformers. These hybrid frameworks leverage the transformer's strong long-sequence modeling capabilities to compensate for CNN's limited receptive field and weak long-range dependencies. They have excellent performance in medical data sets such as brain tumors and organ segmentation. Both Res-Unet and nnUNet are pure CNN-based architectures. Res-Unet introduces residual connections in the encoder-decoder, effectively alleviating the problem of vanishing gradients and making it suitable for small-sample medical image segmentation. nnUNet is an automated medical image segmentation framework. It has achieved excellent performance in multiple medical segmentation challenges and exhibits strong generalization performance. By including pure CNNs models and hybrid models, we can better demonstrate the advantages of our proposed method in the results.

## 3 Results

### 3.1 Implementation details

Our method was implemented on Python 3.9.12 and Pytorch 2.1.1. Experiments were deployed based on the MedNeXt framework, using five-fold cross-validation and the data preprocessing operations built into the MedNeXt framework to perform format conversion, cropping, resampling, and standardization on the data, resulting in a data size of 128 × 128 × 128 with a voxel size of 1mm × 1mm × 1mm. The model training batch size was 2, the initial learning rate was 10^-3^ and the maximum epoch was 100. Frame size configuration was M, kernel size was 3, λ was 0.5 and the optimizer was AdamW.

For all comparison models, the training batch size, patch size and maximum epoch were the same as those of MedNeXt. The initial learning rate was 10^-2^ and the optimizer was SGD.

### 3.2 Results on open and private datasets

First, we use five indicators to evaluate the predictions of various models, showing the overall and subtype results, respectively. Secondly, we compared the prediction results of different models in terms of individual detection rate and slice (axial, sagittal, and coronal) detection rate, enabling a comprehensive analysis of the results. Finally, we present and discuss the 16 negative cases in the Private Dataset separately.

**Open dataset result:** As listed in Table 2, compared to MedNeXet_M3, our method improved the DC metric by 2.40%. LEM-UNet also achieved 2.00% of the DC score over the MedNeXet_L3 which used more convolutional layers. Compared with other CNN-based models, LEM-UNet showed significant improvements in these five metrics. Among transformer-based models, our model exhibited a substantial advantage, with a 5.80% increase in the DC metric over the comparatively high-performing CoTr model. The results indicated that nnFormer and UNETR++ performed poorly in this dataset, struggling to capture the details and characteristic edge of the FCD lesions. In terms of the ability to identify positive class samples, our model excelled, with a notably improved ability to capture FCD lesions details after integrating LEA blocks.

**Table 2:** Performance metrics for different methods on Open and Private datasets.

**Dataset**	**Open dataset**				
**Methods**	**DC**↑	**Pre**↑	**Rec**↑	**IoU**↑	*HD*_95_↓
UNETR++	0.367 ± 0.316	0.472 ± 0.406	0.362 ± 0.331	0.271 ± 0.247	28.189 ± 33.847
CoTr	0.394 ± 0.318	0.466 ± 0.382	0.397 ± 0.319	0.291 ± 0.244	33.997 ± 39.651
nnFormer	0.343 ± 0.275	0.398 ± 0.345	0.376 ± 0.312	0.241 ± 0.207	59.206 ± 40.810
Res-Unet	0.416 ± 0.310	0.528 ± 0.387	0.403 ± 0.324	0.310 ± 0.249	20.451 ± 26.830
nnUNet	0.403 ± 0.316	0.524 ± 0.390	0.384 ± 0.328	0.300 ± 0.253	22.318 ± 29.766
MedNeXt_L3	0.432 ± 0.312	0.522 ± 0.384	0.444 ± 0.338	0.325 ± 0.254	17.525 ± 27.450
MedNeXt_M3	0.428 ± 0.316	0.543 ± 0.391	0.418 ± 0.333	0.323 ± 0.255	20.490 ± 26.986
**LEM-UNet(our)**	**0.452** **±** **0.310**	**0.546** **±** **0.384**	**0.447** **±** **0.338**	**0.346** **±** **0.263**	**16.749** **±** **26.830**
**Dataset**	**Private Dataset**				
UNETR++	0.549 ± 0.269	0.569 ± 0.309	0.621 ± 0.299	0.432 ± 0.238	27.138 ± 21.695
CoTr	0.534 ± 0.246	0.569 ± 0.303	0.590 ± 0.281	0.399 ± 0.209	27.935 ± 22.156
nnFormer	0.512 ± 0.237	0.497 ± 0.286	0.651 ± 0.261	0.376 ± 0.204	35.113 ± 24.957
Res-Unet	0.556 ± 0.238	0.560 ± 0.286	0.604 ± 0.268	0.419 ± 0.212	26.513 ± 20.968
nnUNet	0.560 ± 0.252	0.584 ± 0.291	0.616 ± 0.275	0.428 ± 0.227	25.846 ± 21.736
MedNeXt_L3	0.579 ± 0.239	0.624 ± 0.288	0.631 ± 0.274	0.444 ± 0.221	24.851 ± 20.782
MedNeXt_M3	0.568 ± 0.250	0.616 ± 0.294	0.630 ± 0.277	0.436 ± 0.230	24.535 ± 19.920
**LEM-UNet(our)**	**0.597** **±** **0.230**	**0.626** **±** **0.278**	**0.659** **±** **0.253**	**0.460** **±** **0.217**	**22.763** **±** **18.263**

An upward arrow (↑) indicates that a higher value for the metric corresponds to better model performance, while a downward arrow (↓) signifies that a lower value indicates better performance. Bold values indicate the best performance value for each metric.

**Private dataset result:** As shown in Table 2, all networks achieved a higher DC score compared to the Open dataset. One reason is that each case in this dataset comprised three modalities of raw data (T1, FLAIR, and FDG-PET), providing the base network with a sufficient amount of rich information. However, our network still demonstrated commendable performance, with a 2.90% improvement in DC metrics over MedNeXt_M3. In terms of the ability to identify positive class samples, our network remained more prominent compared to other networks, with a 2.90% increase in Rec over MedNeXt_M3. Secondly, compared to transformer-based networks, LEM-UNet still demonstrated significant advantages, particularly in terms of predictive precision, indicating that our network is more capable of focusing on the subtle details of the lesions.

**Classification results:** In LEM-UNet, the segmentation performance according to histopathology and seizure-free state is shown in Table 3. In the Private Dataset, the DC score for 13 subjects with FCD type I reached 0.655, whereas for 23 subjects with the other MCDs, the DC score was 0.620.

**Table 3:** Segmentation performance based on pathology and seizure freedom in Open and Private dataset.

	**DC↑**	**Rec↑**	**DC>0.0 (n)**	**Patients (n)**
**Private Dataset**				
FCD I	0.655 ± 0.203	0.704 ± 0.236	13	13
FCD IIA	0.605 ± 0.247	0.680 ± 0.233	31	31
FCD IIB	0.555 ± 0.239	0.650 ± 0.281	51	52
FCD IIIB	0.806	0.742	1	1
MCD	0.620 ± 0.217	0.591 ± 0.247	23	23
Other	0.677 ± 0.117	0.789 ± 0.057	5	5
**Seizure freedom**				
Seizure-free	0.600 ± 0.233	0.669 ± 0.250	117	118
Not seizure-free	0.554 ± 0.158	0.444 ± 0.207	7	7
**Open Dataset**				
FCD IIA	0.357 ± 0.349	0.306 ± 0.319	9	15
FCD IIB	0.578 ± 0.254	0.554 ± 0.282	27	31
NA	0.375 ± 0.339	0.410 ± 0.377	21	32
**Seizure freedom**				
Seizure-free	0.538 ± 0.293	0.491 ± 0.296	29	34
Not seizure-free	0.380 ± 0.323	0.362 ± 0.329	5	8
NA	0.386 ± 0.338	0.424 ± 0.383	23	36

“Other” includes TSC, Gliosis, and MOGHE pathologies.

**Detection result:** We analyzed the detection rate results of different models. Table 4 showed the results of the detection rate at the individual and slice level, respectively. LEM-UNet still demonstrated superior performance. One of the major challenges in FCD lesion detection is the occurrence of false-positive clusters that do not have any overlap with the ground truth. Table 5 showed the statistical results on the individual level, which counts the false-positive, true positive, false negative, and ground truth clusters, respectively. All predicted results were subjected to the same connected component post-processing procedure. If retaining only the largest connected component did not yield an improvement in the DC score, the original predictions were preserved, and no non-largest connected components were removed. Independent connected components were identified using the standard 3D 26-connectivity criterion. The results demonstrate that our method exhibits strong performance in identifying true positive clusters, achieving the fewest false-positive clusters on the Open Dataset. In contrast, nnFormer predicted a relatively larger number of false-positive clusters on both datasets.

**Table 4:** Comparison of individual detection rates and average detection rates across the three directional slices between LEM-UNet and other models across two Datasets.

	**Individual**				**Slice**			
**Dataset**	**Open Dataset**		**Private Dataset**		**Open Dataset**		**Private Dataset**	
**Methods**	**DC**>**0.0**	**DC**>**0.22**	**DC**>**0.0**	**DC**>**0.22**	**DC**>**0.0**	**DC**>**0.22**	**DC**>**0.0**	**DC**>**0.22**
UNETR++	64.10	58.97	92.80	87.20	51.97	47.44	76.67	69.62
CoTr	**73.08**	64.10	92.80	84.80	58.49	51.82	76.25	69.04
nnFormer	70.51	62.82	97.60	85.60	55.41	50.25	81.15	70.91
Res-Unet	71.79	66.67	96.00	88.00	58.27	52.15	78.58	70.76
nnUNet	70.51	62.82	96.80	85.60	55.41	50.08	78.90	70.93
MedNeXt_L3	**73.08**	**67.95**	97.60	**91.20**	61.06	56.18	78.63	71.88
MedNeXt_M3	71.79	66.67	96.80	88.80	58.97	53.22	78.32	71.03
**LEM-UNet(our)**	**73.08**	**67.95**	**99.20**	89.60	**61.45**	**56.78**	**81.22**	**74.16**

Bold values indicate the best performance value for each metric.

**Table 5:** Comparisons of the number of ground GT lesions, TP lesions, FP lesions, and FN lesions among models different on two Datasets.

	**TP lesions**	**FP lesions**	**FN lesions**	**GT lesions**
**Private Dataset**				
UNETR++	116	42	9	125
CoTr	116	**9**	9	125
nnFormer	122	101	3	125
Res-Unet	123	31	2	125
nnUNet	121	34	4	125
MedNeXt_L3	122	23	3	125
MedNeXt_M3	121	57	4	125
**LEM-UNet(our)**	**124**	17	**1**	125
**Open Dataset**				
UNETR++	50	45	28	78
CoTr	**57**	78	**21**	78
nnFormer	55	109	23	78
Res-Unet	56	30	22	78
nnUNet	55	37	23	78
MedNeXt_L3	**57**	23	**21**	78
MedNeXt_M3	56	20	22	78
**LEM-UNet(our)**	**57**	**13**	**21**	78

TP, true positive; FP, false-positive; FN, false negative; GT, ground truth. Bold values indicate the best performance value for each metric.

**MRI-negative result:** We included a discussion on MRI-negative patients. Table 6 displayed the results of different models on 16 MRI-negative patients from Private Dataset. These 16 patients appeared positive on PET. Most models experienced varying degrees of performance degradation on these difficult-to-identify MRI-negative patients. For example, the nnUNet's DC score dropped by 6.00%, and the Res-Unet's DC score dropped by 2.00%. However, the hybrid transformer architecture model appeared relatively stable. Our method showed only a slight performance degradation and fully identified all 16 MRI-negative patients.

**Table 6:** The evaluation results of MRI-negative patients on different models.

**Methods**	**DC↑**	**Pre↑**	**Rec↑**	**IoU↑**	***HD*_95_↓**	**DC>0.0**	**FP lesions**
UNETR++	0.551 ± 0.244	0.586 ± 0.301	0.602 ± 0.272	0.415 ± 0.221	32.477 ± 30.586	15	18
CoTr	0.533 ± 0.224	0.559 ± 0.280	0.605 ± 0.298	0.388 ± 0.179	33.176 ± 26.552	15	**2**
nnFormer	0.507 ± 0.210	0.529 ± 0.301	**0.633** **±** **0.211**	0.363 ± 0.176	42.413 ± 30.991	**16**	34
Res-Unet	0.536 ± 0.235	0.572 ± 0.276	0.563 ± 0.287	0.394 ± 0.186	35.872 ± 31.909	14	5
nnUNet	0.500 ± 0.224	0.540 ± 0.289	0.549 ± 0.290	0.360 ± 0.192	35.380 ± 24.813	**16**	11
MedNeXt_L3	0.555 ± 0.248	0.594 ± 0.288	0.594 ± 0.284	0.419 ± 0.224	32.904 ± 31.399	**16**	10
MedNeXt_M3	0.560 ± 0.196	0.613 ± 0.254	0.607 ± 0.233	0.413 ± 0.192	33.912 ± 30.913	**16**	12
**LEM-UNet(our)**	**0.591** **±** **0.199**	**0.673** **±** **0.267**	0.613 ± 0.236	**0.445** **±** **0.193**	**27.542** **±** **26.472**	**16**	3

Bold values indicate the best performance value for each metric.

### 3.3 Visualization comparison and discussion

**Figure 5:**
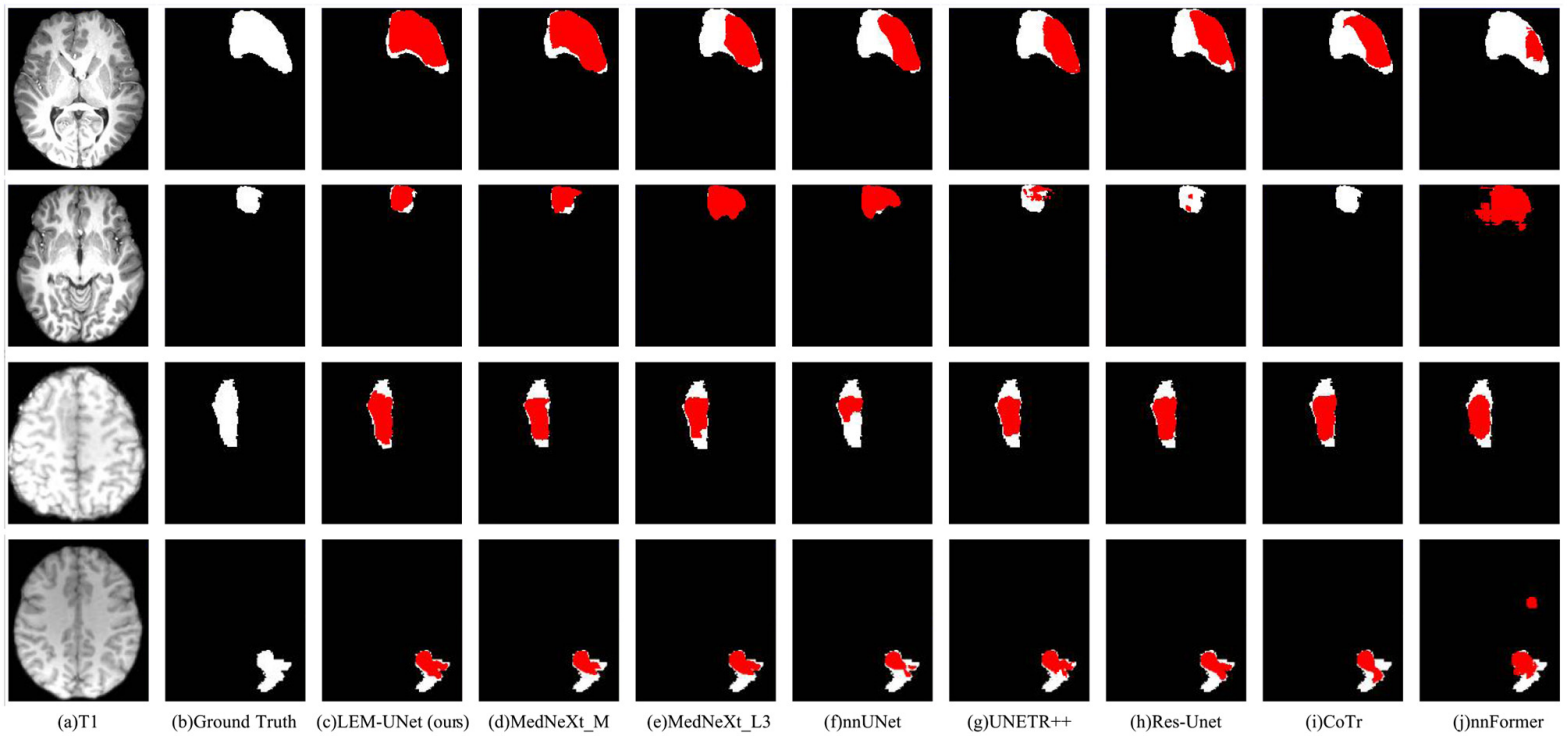
Visualization comparison on Open and Private Datasets (Private Dataset: first two rows, Open Dataset: last two rows). **(a)** The T1 image, **(b)** ground truth, **(c–j)** illustrated the visualization results of LEM-UNet(ours), MedNeXt_M3, MedNeXt_L3, nnUNet, UNETR++, Res-Unet, CoTr, and nnFormer separately.

The visualization results of two datasets are illustrated in Figure 5. The first two rows display the visualization results on the Private Dataset, while the last two rows show the visualization results on the Open Dataset. The ground truth is set to white, while the model's predicted results are set to red and superimposed upon the ground truth for comparison. We observe that transformer-based models exhibit a significantly lower ability to capture positive samples in this task compared to CNN-based models. They struggle to correctly identify FCD lesions with blurred edges and subtle features, even resulting in missed detections, as seen in the CoTr prediction results. By comparing all visualized outcomes, LEM-UNet demonstrates superior performance in capturing the lesion details. Furthermore, our network, by enhancing the focus on edge information, becomes more sensitive to lesion edges, increasing the number of true positive identifications while reducing the likelihood of false positives.

**Figure 6:**
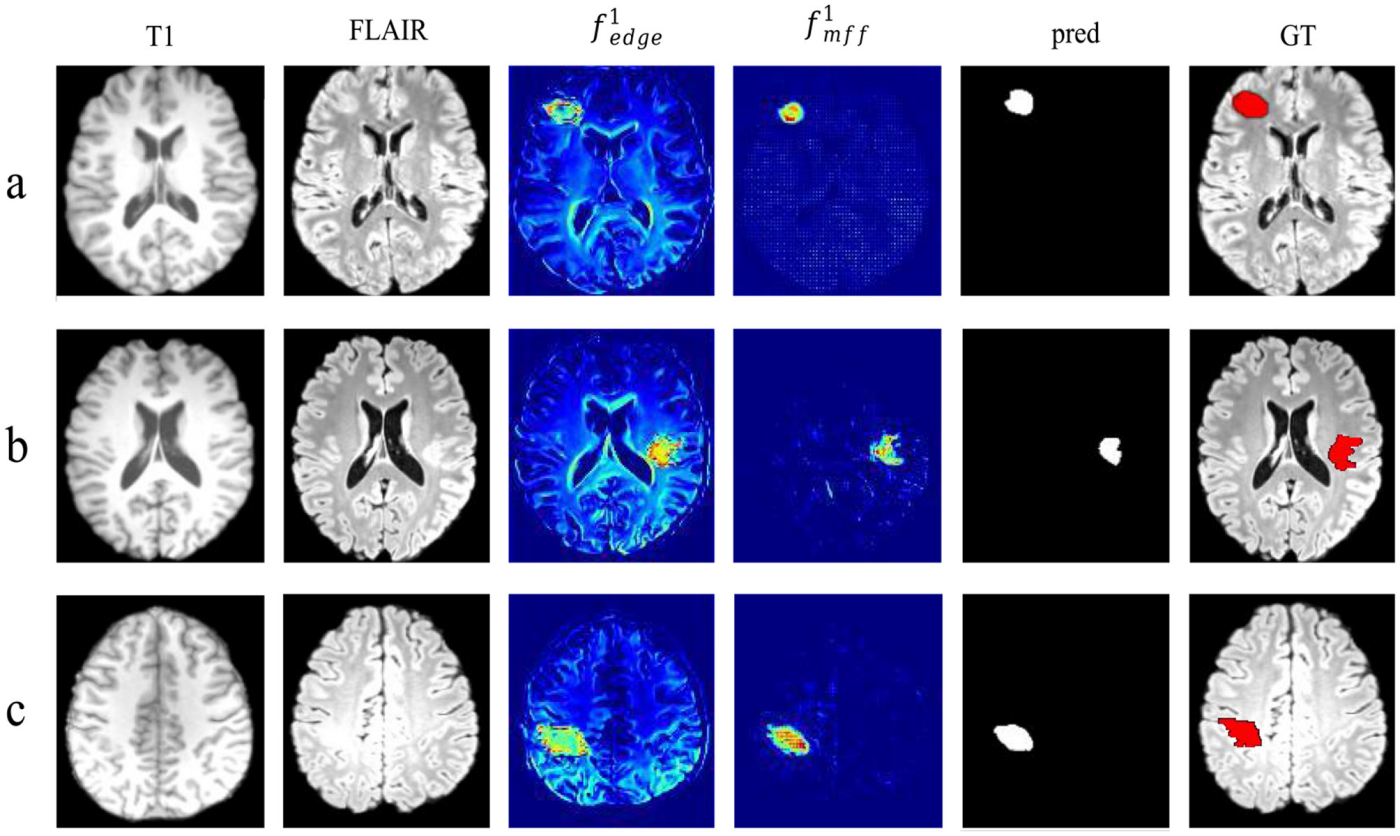
Heatmap visualization of the output in LEA and MFF blocks. Each row represents a case **(a–c)** from Open Dataset. The first column displays the patient's T1 image, the second column shows the patient's FLAIR image, the third column shows the output of the first LEA block: $f_{edge}^{1}$, the fourth column presents the output of the last MFF block: $f_{mff}^{1}$, the fifth column displays the predict result, and the last column depicts the GT that are set to red.

The heat map shown in Figure 6 further illustrates the network focus when processing features. In the LEA block, it has a stronger focus on the edges of the lesion, with a color gradient from blue to red indicating low to high intensity.

### 3.4 Ablation study

In this section, we underscore the significance of each proposed block by conducting a series of ablation studies. Before proceeding with the ablation experiments, a brief discussion will be held on the initialization value of the learnable parameter λ. The experiments will be conducted on the Private Dataset, and the results are shown in Table 7.

**Table 7:** λ initialization in LEM-UNet on Private Dataset.

**λ**	**DC↑**	**Pre↑**	**Rec↑**
0.7	0.589	0.631	0.635
0.5	**0.597**	0.626	**0.659**
0.3	0.591	**0.634**	0.637

Bold values indicate the best performance value for each metric.

Performance is evaluated based on three metrics: DC, Pre, and Rec. It is observed that when λ = 0.5, the model exhibited superior comprehensive performance, with a higher Pre and DC score compared to when λ = 0.3 or λ = 0.7. Next, we proceed with the deployment of ablation studies. Within the context of the LEM-UNet framework, the LEA and MFF blocks are of paramount importance. Given that one of the inputs to the MFF block is the output of the LEA block, this paper evaluates the segmentation outcomes when the MFF and LEA blocks are sequentially removed, in order to validate the performance of each block. We conduct these assessments on the MedNeXt_M3 baseline with a parameter setting of λ = 0.5, using the Private Dataset, and present the results, as detailed in Table 8.

**Table 8:** Ablation study of proposed blocks for LEM-UNet on Private Dataset.

**Baseline**	**LEA block**	**MFF block**	**DC↑**
✓			0.568
✓	✓		0.588
✓	✓	✓	0.597

In LEM-UNet, the incorporation of the edge attention module improves model capabilities but also introduces additional resource overhead. We conducted a comprehensive comparison of the parameter counts and computational complexity between LEM-UNet and other models, as shown in Table 9. Compared to the baseline model MedNeXt_M3, LEM-UNet exhibits higher computational complexity but achieves superior segmentation performance. Our approach strikes a better balance between computational resource utilization and performance enhancement.

**Table 9:** Comparison of computational complexity for different models on Private Dataset.

**Method**	**Parameters (M)**	**GFLOPs**	**DC↑**
UNETR++	42.64	72.52	0.549
CoTr	41.91	689.97	0.534
nnFormer	37.67	63.68	0.512
Res-Unet	12.08	678.97	0.556
nnUNet	31.20	482.69	0.560
MedNeXt_L3	61.78	500.00	0.579
MedNeXt_M3	17.54	254.44	0.568
LEM-UNet (our)	17.66	336.83	0.597

## 4 Discussion

The segmentation of FCD lesions is highly complex, as their characteristics vary across different imaging modalities and are often characterized by blurred edge, posing significant challenges to the segmentation task. The success of FCD lesion resection surgery heavily depends on the precision of the resection area. Therefore, we propose integrating edge enhancement methods into convolutional networks, aiming to improve segmentation performance by enhancing high-frequency edge information of lesions, thereby providing a reliable reference for the clinical diagnosis of FCD lesions.

From the experimental results, LEM-UNet demonstrates superior performance, outperforming comparison models across multiple key metrics, as shown in Table 2. Furthermore, our method exhibits significant advantages in individual and slice detection rates, achieving 99.20% and 81.22% (DC > 0.0) on the Private Dataset, respectively. Based on visualization of feature maps, the LEA block demonstrates the ability to capture high-frequency edge information of lesions, as illustrated in Figure 6, although these edge cues are not entirely closed. According to the ablation study, the integration of the Laplacian pyramid with CBAM effectively enhances the network's focus on high-frequency edge information of FCD lesions, which is one of the key factors contributing to the improved segmentation performance of LEM-UNet.

Deep learning-based methods have demonstrated promising performance in FCD lesion segmentation tasks (e.g., Thomas et al., 2020; Zhang X. et al., 2024; Niyas et al., 2021). However, compared to the excellent segmentation results in other tasks, such as brain tumor segmentation and polyp segmentation, there is still under-segmentation, which may be closely related to the complexity of FCD lesions. Previous approaches primarily focused on feature fusion and enhancement, contributing to addressing segmentation challenges such as semantic gaps, spatial information loss, and limited receptive fields. However, they often overlooked edge features that are particularly meaningful for segmentation tasks. To address this, we introduced the Laplacian pyramid to process lesion edges, leveraging the characteristics of FCD lesions to reduce the false positive rate in segmentation results. By incorporating edge analysis tailored to FCD lesion features, LEM-UNet maximizes the capture of morphological characteristics of lesion edges while focusing on deep abstract features, which is a key advantage of our method over existing algorithms.

Nevertheless, the proposed method still suffers from the inherent limitations of deep learning frameworks, such as its dependence on the accuracy of training data annotations. Additionally, the small dataset size restricts the model's generalizability and robustness. We performed five-fold cross-validation on both Open and Private Datasets and conducted a series of optimization experiments. However, due to the limited availability of FCD data, our method did not use scan data from another center as an independent test set to test the model performance, which is another limitation. Future work will integrate multi-center data to comprehensively enhance the model's generalization capability.

## 5 Conclusion

In this study, we introduce LEM-UNet, an advanced framework for 3D FCD lesions segmentation in multimodal medical images, fortified by the innovative LEA and MFF blocks. We designed the LEA block, which uses a 3D Laplacian pyramid to capture shallow edge information in images, combined with the CBAM to achieve precise localization from global information to local lesion information, further guiding the network to focus on lesion edges and detailed information. Additionally, we designed the MFF block, which combines high-frequency edge features with advanced prediction features, overcoming semantic differences between feature maps while further eliminating redundant information. We deploy these designed modules in the MedNeXt_M3 framework, and the results evaluated through five metrics demonstrate that our method provides competitive performance, with overall metrics superior to other SOTA methods. This emphasizes the effectiveness of our proposed framework in the field of medical image segmentation.

## Data Availability

The datasets presented in this article are not readily available because our Private Dataset is restricted by ethical requirements. Requests to access the datasets should be directed to Open Dataset: https://openneuro.org/datasets/ds004199/.
